# Methionine as a key player in salt stress adaptation in plants

**DOI:** 10.1093/plphys/kiaf169

**Published:** 2025-05-14

**Authors:** Xuelian Li, Ning Zhang

**Affiliations:** State Key Laboratory of Wheat Improvement, College of Agronomy, Shandong Agricultural University, Tai'an, Shandong 271018, China; State Key Laboratory of Wheat Improvement, College of Agronomy, Shandong Agricultural University, Tai'an, Shandong 271018, China

Salt stress is one of the major environmental challenges limiting plant growth and productivity. High salinity leads to osmotic stress, ion toxicity, and oxidative damage (Yang and Guo 2018), which ultimately inhibits plant development and crop yields. Plants have evolved various strategies to mitigate salt stress, including ion homeostasis regulation, osmoprotection, and activation of stress-responsive genes. One of the well-known responses to salt stress is the activation of abscisic acid (ABA) signaling (Barrero et al. 2006; Geng et al. 2013), which plays a crucial role in enhancing stress tolerance by regulating gene expression, ion transport, and redox balance (Park et al. 2009; Yu et al. 2019; Zhang et al. 2021).

Amino acids are also known to participate in stress adaptation. Proline, for instance, is frequently used as a marker for plant stress and has been shown to improve salt tolerance when applied exogenously in various plant species (El Moukhtari et al. 2020). However, recent findings indicate that proline is not the only amino acid involved in salt tolerance. A study revealed that after 138 days of salt treatment on *Chlorella* spp., genes associated with the metabolism of tryptophan, histidine, glycine, serine, and threonine were upregulated, while tyrosine metabolism was downregulated (Li et al. 2018). These observations suggest a broader and more complex role for amino acid metabolism in plant salt stress responses than previously appreciated. Additionally, under saline conditions, the mutation of methionine S-methyltransferase gene (*MMT*), which encodes a key enzyme in methionine biosynthesis, significantly reduces the germination rate and early growth of *Arabidopsis* (Ogawa and Mitsuya 2012). However, the specific function and underlying mechanisms of methionine in salt stress response remained largely unexplored.

In this issue of *Plant Physiology*, Shi et al. (2025) wondered whether methionine contributes to plant salt tolerance, as the germination rate and early growth of *mmt* mutants were significantly inhibited under salt treatment. They found that exogenous methionine application significantly improved salt stress resistance in *Arabidopsis*, soybean (*Glycine max*), and maize (*Zea mays*). The observed tolerance was linked to increased proline accumulation, the well-known osmoprotectant that stabilizes cellular osmotic balance and turgor pressure (Khedr et al. 2003). Furthermore, plants treated with methionine exhibited lower levels of malondialdehyde, an indicator of oxidative damage. Collectively, these results suggested that methionine contributes to plant salt tolerance.

Next, the authors investigated whether salt stress influences methionine levels in plants by modulating the expression of methionine biosynthesis genes. In response to salt stress, the expression of key genes involved in methionine biosynthesis, particularly *Hcy-S-methyltransferases* (*HMT*s) and *methionine synthases* (*MS*s), was significantly upregulated, leading to increased methionine accumulation. Phenotypic analysis of methionine biosynthesis mutants further reinforced this finding. These mutants exhibited significantly shorter roots and lower survival rates compared to the wild-type control under salt stress, indicating the crucial role of methionine in maintaining root architecture and stress resilience.

Given the roles of ABA and ROS as key signaling molecules in plant growth regulation and salt stress responses, the authors next explored their interplay with methionine biosynthesis. They found that both ABA and ROS were required for the upregulation of methionine biosynthesis genes under salt stress. Further analysis revealed that methionine enhances plant salt tolerance by activating the ABA pathway. This was confirmed through transcriptome analysis, which showed that methionine treatment rapidly activated ABA biosynthetic and signaling genes, leading to higher endogenous ABA levels (Figure). Also, post-germination growth analysis showed that methionine strengthens ABA-induced inhibition of cotyledon development, fresh weight, and root growth. Moreover, the positive effect of methionine on salt tolerance was significantly reduced in ABA-related mutants, indicating methionine activates ABA signaling and the regulation of salt stress–responsive genes to improve stress tolerance.

**Figure.**
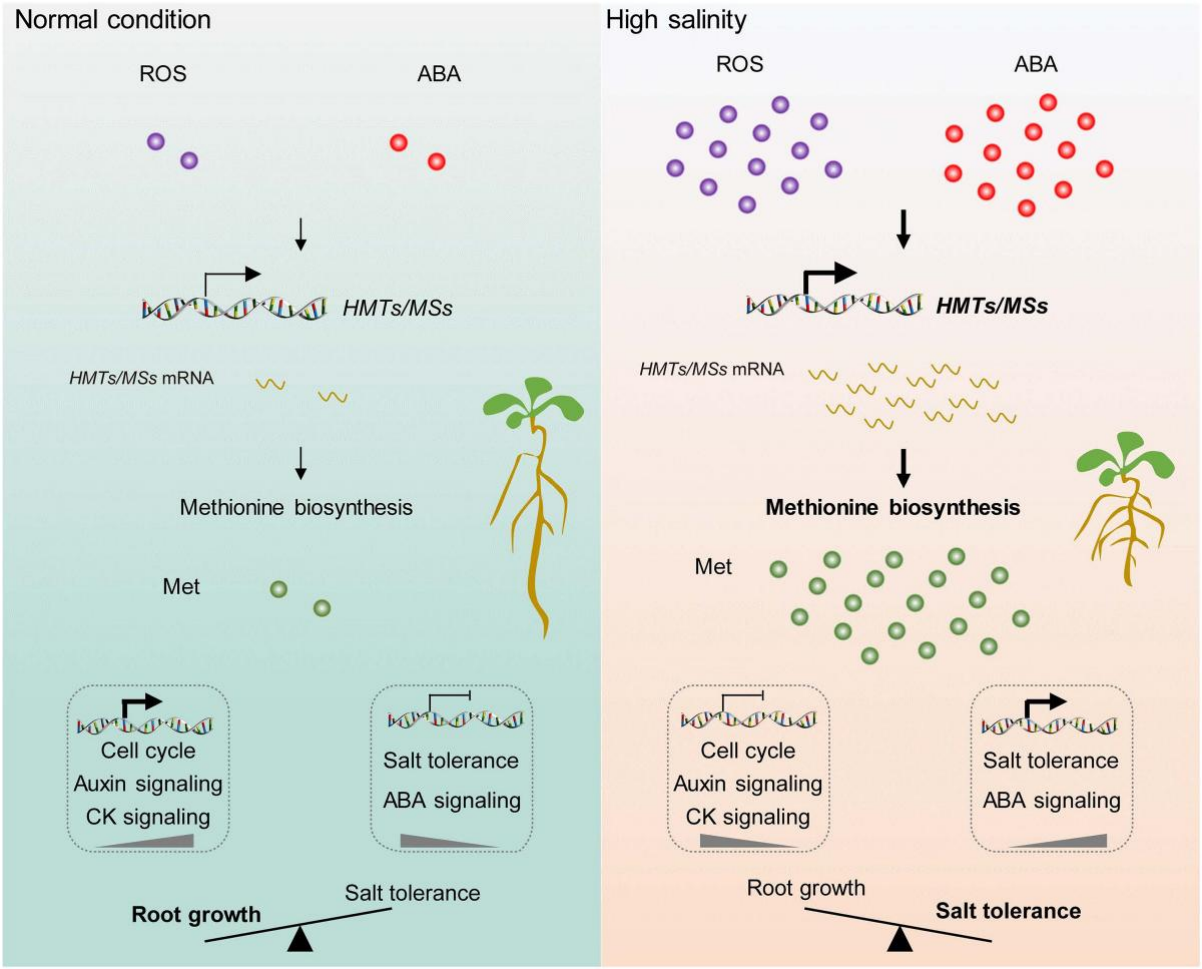
A schematic illustration of the methionine-mediated balance between salt tolerance and root growth. Under normal conditions, low levels of ROS and ABA signaling maintain low methionine synthesis, promoting root growth through activating cell cycle, auxin, and cytokinin pathways while repressing salt tolerance genes. In contrast, high salinity activates ROS and ABA signaling, upregulates methionine biosynthesis, and elevates methionine levels. This shift enhances salt tolerance by boosting ABA signaling and salt-responsive gene expression, while simultaneously inhibiting root growth by suppressing genes involved in cell division and hormone signaling (Figure from Shi et al. 2025).

The authors further found that methionine acted as a regulatory switch between primary root growth and stress adaptation. It inhibited primary root elongation in a dose-dependent manner, partly through repression of cell cycle progression and hormone signaling pathways, specifically auxin and cytokinin. Although methionine is a precursor for ethylene, its effects on root growth inhibition were independent of ethylene signaling, suggesting a distinct regulatory role for methionine. This methionine-mediated trade-off ensures that plants can effectively balance growth and stress adaptation under high salinity conditions.

This study provides compelling evidence that methionine plays a dual role in plant salt stress adaptation by activating ABA signaling to enhance stress tolerance while simultaneously suppressing root growth to optimize energy allocation (Fig. 1). Although the study did not explicitly investigate a feedback loop between ABA and methionine, the findings raise interesting questions about their possible reciprocal regulation. ABA signaling was shown to enhance methionine biosynthesis by upregulating key genes involved in its production, suggesting a role for ABA in modulating methionine levels under salt stress. At the same time, methionine treatment increased endogenous ABA levels and activated ABA-responsive genes, indicating that methionine may influence ABA biosynthesis and signaling. How ABA and methionine maintain homeostasis under salt stress remains an open question. Given their mutual influence, a finely balanced mechanism may exist to regulate their levels and prevent excessive responses that could be detrimental to plant growth. Future studies could explore whether plants have built-in regulatory controls to adjust ABA and methionine concentrations dynamically.

## Data Availability

There are no new data associated with this article.
